# Case Report: Combined Intercostal-Transdiaphragmatic-Abdominal Wall Hernia

**DOI:** 10.3389/jaws.2025.14535

**Published:** 2025-07-04

**Authors:** P. Martínez-López, M. Verdaguer-Tremolosa, V. Rodrigues-Gonçalves, A. Martín-Del-Rey, M. López-Cano

**Affiliations:** ^1^ Department of Surgery, UD of Medicine of Vall d’Hebron, Universitat Autònoma de Barcelona, Barcelona, Spain; ^2^ Abdominal Wall Surgery Unit, General and Digestive Surgery Department, Hospital Universitari Vall d’Hebrón, Barcelona, Spain; ^3^ General and Digestive Surgery Department, Hospital Universitari Vall d’Hebrón, Barcelona, Spain

**Keywords:** intercostal hernia, transdiaphragmatic hernia, abdominal wall hernia, chronic obstructive pulmonary disease, cough

## Abstract

**Aim:**

To present a rare and complex case of a spontaneous intercostal, transdiaphragmatic and abdominal wall hernia in an elderly male with a history of chronic obstructive pulmonary disease (COPD).

**Methods:**

According to the CARE checklist, we describe a rare case of intercostal, transdiaphragmatic and abdominal wall hernia after an episode of severe coughing.

**Results:**

A 72-year-old male presented with nausea, dyspnea, and progressive left thoracic and abdominal swelling, along with a history of severe cough and spontaneous hematoma in the same regions. A CT scan revealed an intercostal hernia between the 8th and 9th ribs, with transdiaphragmatic extension and involvement of the lateral abdominal wall, containing most of the stomach, transverse colon, splenic flexure, descending colon, and small intestine. An elective left thoraco-abdominal open surgery was performed, including preperitoneal hernioplasty with dual mesh placement and repair of the diaphragmatic and costal defect.

**Conclusion:**

Such cases are scarcely reported in the literature. This case highlights the importance of considering complex hernia in patients with severe COPD and the importance of early treatment along with a multidisciplinary surgical approach.

## Introduction

Intercostal, transdiaphragmatic, and abdominal wall hernias are exceptionally rare due to the extensive nature of the hernia and the involvement of multiple compartments, including thoracic and abdominal cavities [1]. When the abdominal viscera gain entry to the intercostal space through an associated diaphragmatic defect, the term of transdiaphragmatic intercostal hernia is usually employed, where as if no diaphragmatic abnormality is present, the term abdominal intercostal hernia (AIH) is used [2, 3]. These types of hernia are almost always located inferiorly to the ninth rib and are predominantly found on the left thoracic side [3, 4]. They usually occur after trauma, but less frequently, increased intrathoracic pressure during episodes of severe coughing can cause a hernia, although this is rarely reported [2].

## Case Description

We report the case of a 72-year-old male, former smoker, with a medical history of arterial hypertension, diabetes mellitus, and chronic obstructive pulmonary disease (COPD). He was hospitalized in December 2023 for community-acquired pneumonia.

**FIGURE 1:**
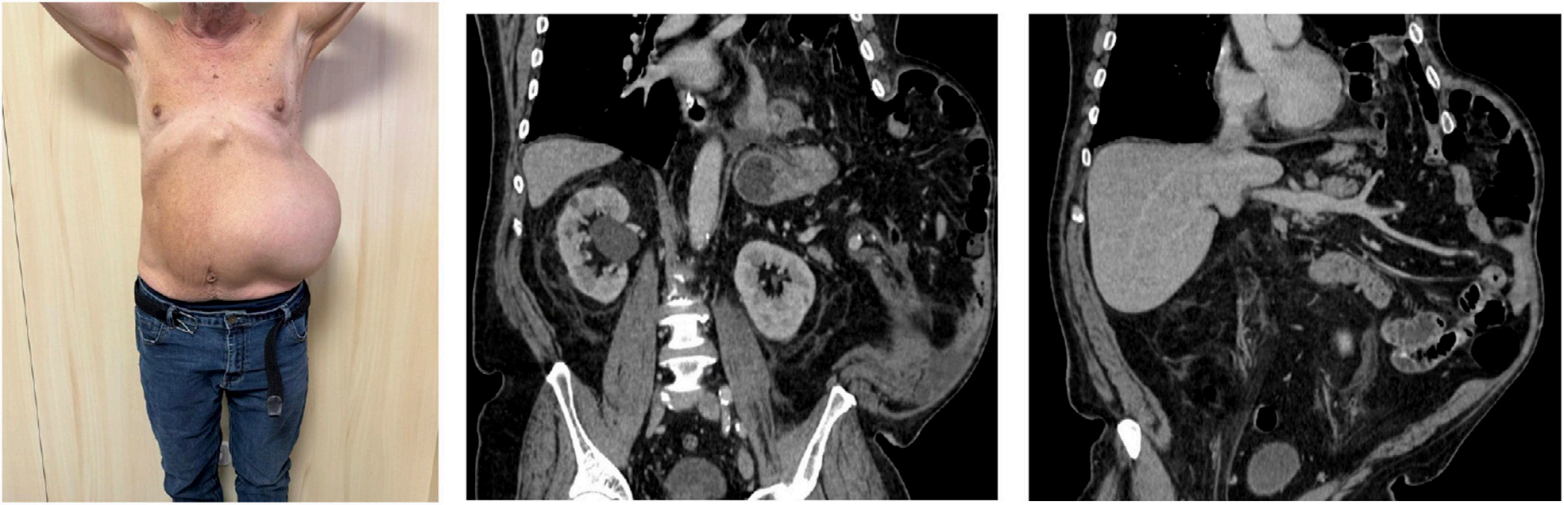
Patient and preoperative CT scan.

In February and March 2024, the patient presented to the emergency department with symptoms including nausea, dyspnea, and progressively increasing left-sided thoracic and abdominal swelling. He reported experiencing a severe cough following the pneumonia episode, which was soon followed by the spontaneous appearance of a hematoma on the left hemithorax and hemiabdomen. On physical examination, significant left-sided thoracic and abdominal swelling was observed, along with clinical signs suggestive of a partially reducible hernia (Figure 1).

A computed tomography (CT) scan revealed an intercostal hernia between the eighth and ninth ribs, extending transdiaphragmatically and involving the left lateral abdominal wall. The hernia sac contained the majority of the stomach, colon and loops of small bowel (Figure 1). The patient underwent preoperative optimization, including prehabilitation focused on weight loss and control of COPD to minimize the risk of hernia exacerbation.

In April 2024, the patient underwent an elective open thoracoabdominal hernia repair. Intraoperative findings included a 9 × 9 cm defect in the internal oblique and transversus abdominis muscles, with costal cartilage disinsertion between the 8th and 9th ribs, as well as an 8 cm diaphragmatic defect. The hernia sac extended into the left abdominal wall, intrathoracic, and intercostal regions.

**FIGURE 2:**
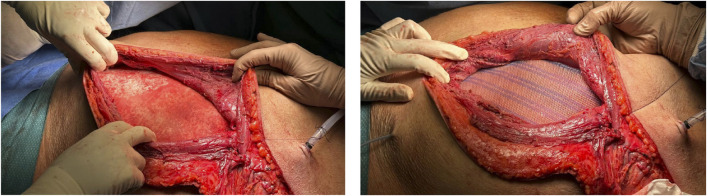
Preperitoneal hernioplasty (Bio-A and polypropylene mesh).

**FIGURE 3:**
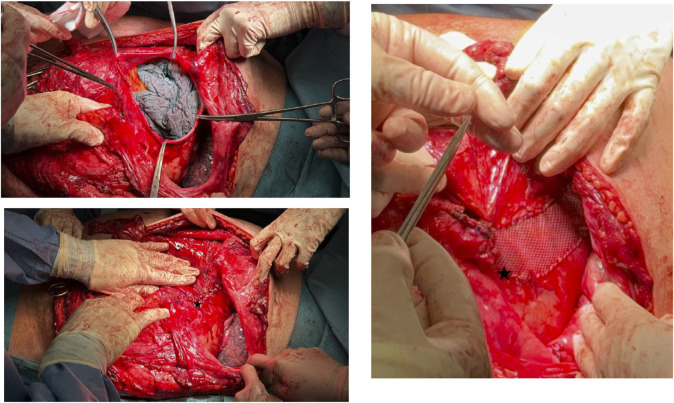
Closure of the diaphragmatic defect with reinforcement mesh.

A preperitoneal hernioplasty was performed using Bio-A and polypropylene mesh to reinforce the abdominal wall (Figure 2). The diaphragmatic defect was repaired and reinforced with a Synecor mesh (Figure 3). Thoracic surgeons closed the intercostal space and placed a thoracic drain, ensuring closure of the communication between the preperitoneal lateral space and the thoracic cavity.

During hospitalization, an episode of renal insufficiency exacerbation occurred, which was successfully managed with targeted therapy. He was discharged after 11 days with no further complications.

At 6-month follow-up, the only significant finding was a minor subcutaneous seroma at the site of the thoracic incision. There were no reports of abdominal or thoracic discomfort, dyspnea, or other complications. The patient continued respiratory rehabilitation, showing effective management of his COPD.

## Discussion

This is a rare pathology in clinical practice, and much of the existing literature consists of case reports. Systematic reviews on this topic highlight the absence of a standardized classification system to guide the management of the various types of hernias, given the diversity in their presentations [5, 6]. These hernias are typically classified as either acquired or spontaneous [1–3, 7, 8]. Acquired hernias are generally secondary to major trauma, such as penetrating injuries, falls, or crush injuries [5], as well as to minor trauma, such as a sudden increase in thoracic pressure caused by severe coughing, childbirth, physical exertion, or vomiting [1–4, 7, 9, 10]. However, in spontaneous cases, the literature reveals that patients often report chronic coughing related to smoking, COPD, obesity, advanced age, or collagenopathies [1, 2, 7, 11, 12]. It remains debatable whether these cases are truly spontaneous or result from repeated minor trauma over time. In our view, this type of hernia should always be considered acquired, either through an acute traumatic event or through chronic, repeated minor trauma from increases in thoraco-abdominal pressure.

The lack of consistency in the literature complicates the identification and management of these cases. In 1978, Le Neel et al. described four cases of what they termed “abdominal intercostal hernia,” defining it as the protrusion of abdominal viscera through an intercostal space following diaphragmatic herniation and intercostal muscle rupture [11]. Since then, this term has been used globally, often without specifying the anatomical areas involved. Gooseman et al. introduced the Sheffield classification, which considers the involvement of costal margin rupture, diaphragmatic rupture, and intercostal rupture [6]. More recently, Byers et al. analyzed management strategies based on this classification, distinguishing between conservative and surgical approaches [5]. Patients with isolated costal margin rupture often benefit from conservative management, with favorable outcomes, while diaphragmatic and/or intercostal ruptures typically require surgical repair [5]. We propose that the involvement of abdominal wall musculature should be integrated into the classification system, as this component is frequently overlooked but crucial in cases like the one presented. Proper management in such cases extends to abdominal wall repair.

A comprehensive, standardized classification system is critical to optimize the management of this pathology and achieve better clinical outcomes. Conventional CT and 3D imaging are generally employed to detect all anatomical defects [5]. While chest X-rays and ultrasound may also be used, they are less effective [13].

The surgical approach is not yet standardized, but it is essential to repair all components of the defect, including the diaphragm, intercostal muscles, abdominal wall, and costal margin, to minimize recurrence and postoperative pain. Open surgery via thoracic or thoracoabdominal incision remains the most common approach [5]. However, there are reports of successful laparoscopic repair, offering early mobilization and minimal postoperative discomfort, though with limited long-term follow-up [4, 14]. Early surgical intervention in scheduled cases can prevent complications such as incarceration, which significantly increases morbidity and mortality [10, 15, 16].

In conclusion, early surgical intervention is key to preventing complications. This case illustrates a rare and complex hernia that can develop in patients with severe COPD, especially after intense coughing. In this case, the severe post-pneumonia cough likely caused the rupture of the diaphragm and abdominal wall, leading to herniation. The strengths of this case include a successful multidisciplinary surgical approach and a favorable postoperative outcome. However, the patient’s underlying pulmonary condition presents a risk of hernia recurrence. This case adds to the limited body of literature on such hernias, underscoring the importance of meticulous preoperative planning and postoperative management in similar cases.

## Data Availability

The datasets presented in this article are not readily available because data about the patient is confidential. Requests to access the datasets should be directed to the local informatic health care system.
